# Ten recommendations for software engineering in research

**DOI:** 10.1186/2047-217X-3-31

**Published:** 2014-12-04

**Authors:** Janna Hastings, Kenneth Haug, Christoph Steinbeck

**Affiliations:** Cheminformatics and Metabolism, European Molecular Biology Laboratory – European Bioinformatics Institute, Wellcome Trust Genome Campus, CB10 1SD Hinxton, UK

**Keywords:** Software engineering, Best practices

## Abstract

Research in the context of data-driven science requires a backbone of well-written software, but scientific researchers are typically not trained at length in software engineering, the principles for creating better software products. To address this gap, in particular for young researchers new to programming, we give ten recommendations to ensure the usability, sustainability and practicality of research software.

## Background

Scientific research increasingly harnesses computing as a platform [1], and the size, complexity, diversity and relatively high availability of research datasets in a variety of formats is a strong driver to deliver well-designed, efficient and maintainable software and tools. As the frontier of science evolves, new tools constantly need to be written; however scientists, in particular early-career researchers, might not have received training in software engineering [2], thus their code is in jeopardy of being difficult and costly to maintain and re-use.

To address this gap, we have compiled ten brief software engineering recommendations.

## Recommendations

### Keep it simple

Every software project starts somewhere. A rule of thumb is to *start as simply as you possibly can*. Significantly more problems are created by over-engineering than under-engineering. Simplicity starts with design: a clean and elegant data model is a kind of simplicity that leads naturally to efficient algorithms.

Do the simplest thing that could possibly work, and then double-check it really does work.

### Test, test, test

For objectivity, large software development efforts assign different people to test software than those who develop it. This is a luxury not available in most research labs, but there are robust testing strategies available to even the smallest project.

Unit tests are software tests which are executed automatically on a regular basis. In test *driven* development, the tests are written first, serving as a specification and checking every aspect of the intended functionality as it is developed [3]. One must make sure that unit tests exhaustively simulate *all possible* – not only that which seems reasonable – inputs to each method.

### Do not repeat yourself

Do not be tempted to use the copy-paste-modify coding technique when you encounter similar requirements. Even though this seems to be the simplest approach, it will not remain simple, because important lines of code will end up duplicated. When making changes, you will have to do them twice, taking twice as long, and you may forget an obscure place to which you copied that code, leaving a bug.

Automated tools, such as Simian [4], can help to detect and fix duplication in existing codebases. To fix duplications or bugs, consider writing a library with methods that can be called when needed.

### Use a modular design

Modules act as building blocks that can be glued together to achieve overall system functionality. They hide the details of their implementation behind a public interface, which provides all the methods that should be used. Users should code – and test – to the interface rather than the implementation [5]. Thus, concrete implementation details can change without impacting downstream users of the module. Application programming interfaces (APIs) can be shared between different implementation providers.

Scrutinise modules and libraries that already exist for the functionality you need. Do not rewrite what you can profitably re-use – and do not be put off if the best candidate third-party library contains more functionality than you need (now).

### Involve your users

Users know what they need software to do. Let them try the software as early as possible, and make it easy for them to give feedback, via a mailing list or an issue tracker. In an open source software development paradigm, your users can become co-developers. In closed-source and commercial paradigms, you can offer early-access beta releases to a trusted group.

Many sophisticated methods have been developed for user experience analysis. For example, you could hold an interactive workshop [6].

### Resist gold plating

Sometimes, users ask for too much, leading to feature creep or “gold plating”. Learn to tell the difference between essential features and the long list of wishes users may have. Prioritise aggressively with as broad a collection of stakeholders as possible, perhaps using “game-storming” techniques [7].

Gold plating is a challenge in all phases of development, not only in the early stages of requirements analysis. In its most mischievous disguise, just a little something is added in every iterative project meeting. Those little somethings add up.

### Document everything

Comprehensive documentation helps other developers who may take over your code, and will also help you in the future. Use code comments for in-line documentation, especially for any technically challenging blocks, and public interface methods. However, there is no need for comments that mirror the exact detail of code line-by-line.

**Figure 1:**
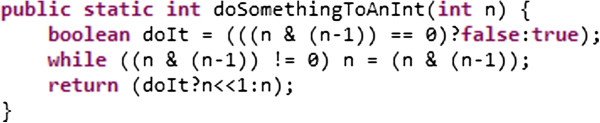
**An example of incomprehensible code: What does this code actually do? It contains a bug; is it easy to spot?**

It is better to have two or three lines of code that are easy to understand than to have one incomprehensible line, for example see Figure 1.

**Figure 2:**
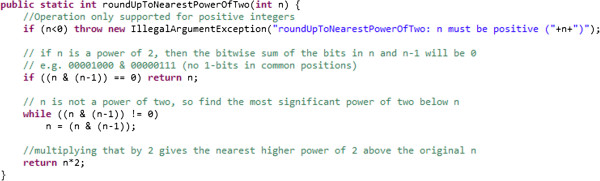
**This code peforms the same function, but is written more clearly.**

Write clean code [8] that *you* would *want* to maintain long-term (Figure 2). Meaningful, readable variable and method names are a form of documentation.

Write an easily accessible module guide for each module, explaining the higher level view: what is the purpose of this module? How does it fit together with other modules? How does one get started using it?

### Avoid spaghetti

**Figure 3:**
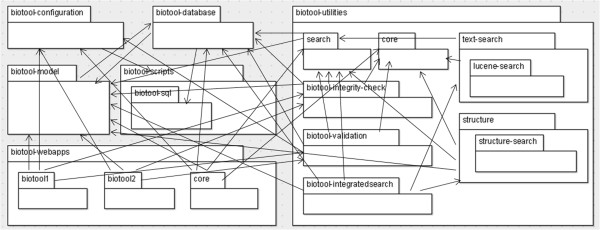
**An unhealthy module design for ‘biotool‘ with multiple interdependencies between different packages.** An addition of functionality to the system (such as supporting a new field) requires updating the software in many different places. Refactoring into a simpler architecture would improve maintainability.

**Figure 4:**
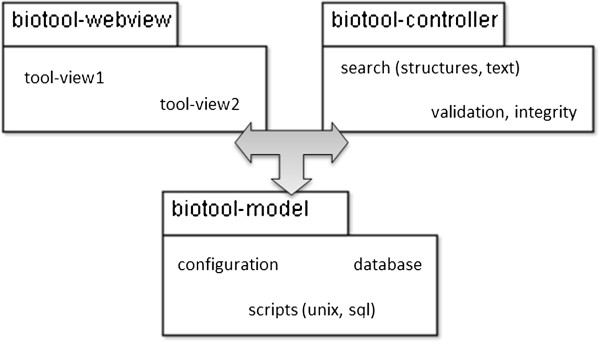
**The functional units from the biotool architecture can be grouped together in a refactoring process, putting similar functions together.** The result may resemble a Model-View-Controller architecture.

Since GOTO-like commands fell justifiably out of favour several decades ago [9], you might believe that spaghetti code is a thing of the past. However, a similar phenomenon may be observed in inter-method and inter-module relationships (see Figures 3 and 4). Debugging – stepping through your code as it executes line by line – can help you diagnose modern-day spaghetti code. Beware of module designs where for every unit of functionality you have to step through several different modules to discover where the error is, and along the way you have long lost the record of what the original method was actually doing or what the erroneous input was. The use of effective and granular logging is another way to trace and diagnose problems with the flow through code modules.

### Optimise last

Beware of optimising too early. Although research applications are often performance-critical, until you truly encounter the wide range of inputs that your software will eventually run against in the production environment, it may not be possible to anticipate where the real bottlenecks will lie. Develop the correct functionality first, deploy it and then continuously improve it using repeated evaluation of the system running time as a guide (while your unit tests keep checking that the system is doing what it should).

### Evolution, not revolution

Maintenance becomes harder as a system gets older. Take time on a regular basis to revisit the codebase, and consider whether it can be renovated and improved [10]. However, the urge to rewrite an entire system from the beginning should be avoided, unless it is really the only option or the system is very small. Be pragmatic [11] – you may never finish the rewrite [12]. This is especially true for systems that were written without following the preceding recommendations.

Use a good version control system (e.g., Git [13]) and a central repository (e.g., GitHub [14]). In general, commit early and commit often, and not only when refactoring.

## Conclusion

Effective software engineering is a challenge in any enterprise, but may be even more so in the research context. Among other reasons, the research context can encourage a rapid turnover of staff, with the result that knowledge about legacy systems is lost. There can be a shortage of software engineering-specific training, and the “publish or perish” culture may incentivise taking shortcuts.

The recommendations above give a brief introduction to established best practices in software engineering that may serve as a useful reference. Some of these recommendations may be debated in some contexts, but nevertheless are important to understand and master. To learn more, Table 1 lists some additional online and educational resources.

**Table 1:** **Further reading**

Description	URL
Software Carpentry: scientific	
computing skills; learn online	
or in face-to-face workshops	http://software-carpentry.org/
The Software Sustainability Institute	http://software.ac.uk/
Learn more about what makes	
code easy to maintain	http://www.thc.org/root/phun/unmaintain.html
How to write good unit tests	http://developer.salesforce.com/page/How_to_Write_Good_Unit_Tests
What is clean code?	http://java.dzone.com/articles/what-clean-code-%E2%80%93quotes
Introduction to refactoring	http://sourcemaking.com/refactoring/
The danger of prematureoptimization	http://c2.com/cgi/wiki?PrematureOptimization

This table lists additional online resources where the interested reader can learn more about software engineering best practices in the research context.
